# Supplementary material for: Don’t dumb it down: The effects of jargon in COVID-19 crisis communication
Source: PLoS One. 2020 Oct 7;15(10):e0239524. doi: 10.1371/journal.pone.0239524 (PMC7540871; doi:10.1371/journal.pone.0239524)
Supplement: S1 Table — (DOCX) [file pone.0239524.s001.docx]

| **COVID-19 (High Urgency)** | **Flood Risk (Low Urgency)** | **Emergency Policy (No Urgency)** |
| --- | --- | --- |
| The United States is preparing for an outbreak of respiratory disease caused by a novel (new) coronavirus that has now been detected in more than 100 countries, including in the U.S. The virus has been named “SARS-CoV-2” and the disease it causes has been named “coronavirus disease 2019” (abbreviated “COVID-19”). On the next page you will receive more detailed information about this type of disease along with guidelines for how to protect yourself during this public health emergency. **(word count = 78)** | The United States is preparing for a new season of natural disasters caused by melting snow and heavy rain. These events are common across the world and in every state in the United States. These natural hazards are called “hydraulic exchange flows” and the disasters they cause are called “floods.” On the next page you will receive more detailed information about this type of disaster along with guidelines for how to protect yourself from this public health emergency. **(word count = 78)** | The United States is always preparing for natural disasters like hurricanes, tornadoes, earthquakes, tsunamis, volcanoes, and floods. These events are common across the world and in every state in the United States. The U.S. policy that determines response to these events is called the “National Response Framework,” or the “NRF.” On the next page you will receive more detailed information about these types of policies along with guidelines for how to protect yourself from a public health emergency. **(word count = 78)** |
| **Jargon Condition**  Note: Jargon words are only underlined here for emphasis, they were not underlined in the experimental stimuli | | |
| SARS-coV-2, a novel coronavirus from the family Coronaviridae, is a zoonotic pathogen and causative agent for COVID-19. COVID-19 is an infectious disease that causes inflammation of the respiratory tract and bronchial tubes. COVID-19 is transmitted through respiratory aerosols or secretions on contaminated surfaces and has an incubation period of 2 to 14 days. During this time individuals may be asymptomatic. According to medical virologists, COVID-19 has the highest lethality for people who are immunocompromised. Epidemiologists from the WHO and the CDC have made a global risk assessment that COVID-19 is a new pandemic and recommend that communities engage in a mitigation phase of disease control. **(word count = 105)** | Hydraulic exchange flows occur when the topography of an area that is usually above water becomes saturated and submerged. These events are caused by excessive precipitation, tidal surges, or ice jams that lead to downstream overbank flow. Hydraulic exchange flow is dangerous for densely populated areas, because the land is impervious and water cannot dissipate. In this case, supplies get contaminated, which threatens habitability in these areas. Individuals whose plumbing systems rely on sump pumps or backflow valves are also at risk of being trapped or swept away. According to the hydrologists from NOAA and FEMA, these events can disrupt transportation and cause widespread erosion. **(word count = 105)** | The NRF determines how the United States responds to major adverse events. The NRF and its support annexes provide guidance about hazards and define roles for inter-agency and inter-organization collaboration. NRF focuses on maintaining community lifeline stabilization and resilient capabilities for hazard identification and risk assessment and is used by critical infrastructure sector leadership to help follow the United States’ national security strategy. The NRF describes how communities benefit from integrated incident response. For voluntary organizations and insular areas, the NRF helps the private sector, NGOs and SMBs conduct a tiered response to support cross-sector operations when critical infrastructure is threatened to reduce cascading failures. **(word count = 105)** |
| To stay protected, experts recommend taking the following precautions.   - Individuals should maintain good hygiene and keep a supply of ethanol-based sanitizers and decontaminants that are certified for emerging viral pathogens claims and can be used on high-touch surfaces. - Individuals should be prepared to shelter in place, engage in social distancing or quarantine, and be able to telecommute. - Keep non-perishable supplies, including items with electrolytes like UHT milk. - Visit an automated teller machine or financial institution and gather vital documents, like immunization records and other EHRs. - Pay attention to advice from FEMA and follow their guidelines about community NPIs.   **(word count = 98)** | | |
| **No Jargon Condition** | | |
| Severe acute respiratory syndrome coronavirus 2, a new virus, is a germ transmitted from animals to humans that causes coronavirus. Coronavirus causes swelling in the nose, throat, and lungs. Coronavirus spreads through droplets of fluid from the eyes, mouth, or nose, and takes 2 to 14 days after getting the infection to show symptoms. During this time individuals may not show signs of disease. Doctors who study viruses say that coronavirus is riskiest for people who are already sick. Scientists from the World Health Organization and Centers for Disease Control think coronavirus will spread and recommend that communities reduce the damaging effects of the disease. **(word count = 105)** | Floods occur when dry land becomes covered with water. Floods are caused by heavy rain, high tides, or melting ice that causes water to flow over the banks of bodies of water. Floods are dangerous in cities because pavement cannot absorb water. In this case, supplies get dirty making it hard for people to live in these areas. Individuals whose plumbing systems rely on pumps that remove water from basements or protect drinking water are at risk of being trapped or swept away. According to scientists from the National Oceanic and Atmospheric Administration and Federal Emergency Management Agency, floods disrupt transportation and cause widespread damage. **(word count = 105)** | The National Response Framework determines how the United States responds to catastrophes like natural disasters. The National Response Framework and its supporting documents provide guidance about natural disasters and help with collaboration between government agencies and organizations. The framework focuses on maintaining utility services like power and water during a disaster and is used by utility company leaders to help follow the United States’ national security strategy. The framework describes how communities benefit from working together in a disaster. For volunteer organizations and rural areas, the framework says that corporations, non-profits, and small businesses should work together to reduce threats during a natural disaster event. **(word count = 105)** |
| To stay protected, experts recommend taking the following steps.   - Stay clean and keep alcohol-based sanitizers and cleaning supplies that are certified for dangerous germs to use on items that are touched a lot. - Be prepared to stay at home, avoid crowds or stay isolated, and work from home. - Keep food that can be stored for a long time, including items with nutrients like ultra-pasteurized milk. - Visit an ATM or bank and gather birth certificates, shot records, and other electronic health records. - Pay attention to advice from the Federal Emergency Management Agency and follow guidelines about community nonpharmaceutical interventions.   **(word count = 98)** | | |
