# Supplementary material for: Don’t dumb it down: The effects of jargon in COVID-19 crisis communication
Source: PLoS One. 2020 Oct 7;15(10):e0239524. doi: 10.1371/journal.pone.0239524 (PMC7540871; doi:10.1371/journal.pone.0239524)
Supplement: S2 Table — (DOCX) [file pone.0239524.s002.docx]

**S2 Table. Descriptive Statistics Across Conditions for All Study Variables.**

|  | Topic | | | | | | | | | |
| --- | --- | --- | --- | --- | --- | --- | --- | --- | --- | --- |
|  | COVID-19 | | | Flood Risk | | | Emergency Policy | | | |
|  | No-Jargon  *M* (SD) | Jargon  *M* (SD) | Total  *M* (SD) | No-Jargon  *M* (SD) | Jargon  *M* (SD) | Total  *M* (SD) | No-Jargon  *M* (SD) | Jargon  *M* (SD) | Total  *M* (SD) |  |
| *N* | 67 | 67 | 134 | 67 | 64 | 131 | 62 | 66 | 128 |  |
| Fluency | 5.22 (1.37) | 5.32 (1.26) | 5.27 (1.31) | 4.95 (1.17) | 4.48 (1.40) | 4.72 (1.31) | 5.28 (1.08) | 4.53 (1.27) | 4.90 (1.24) |  |
| MRTP | 2.93 (1.14) | 2.42 (1.19) | 2.67 (1.19) | 3.05 (1.30) | 3.18 (1.15) | 3.11 (1.23) | 2.72 (0.94) | 2.90 (1.19) | 2.81 (1.08) |  |
| Cred. | 6.10 (0.78) | 6.41 (0.65) | 6.25 (0.73) | 5.32 (1.42) | 5.56 (1.12) | 5.43 (1.29) | 5.87 (0.92) | 5.97 (0.68) | 5.92 (0.80) |  |
| Risk | 5.85 (1.14) | 6.34 (0.82) | 6.09 (1.02) | 5.24 (1.27) | 5.47 (1.15) | 5.35 (1.22) | 6.03 (0.93) | 6.06 (0.86) | 6.05 (0.89) |  |
| Severity | 3.93 (1.54) | 4.15 (1.42) | 4.04 (1.48) | 3.57 (1.32) | 3.65 (1.67) | 3.61 (1.49) | 4.66 (1.00) | 4.73 (1.16) | 4.70 (1.08) |  |
