# Supplementary material for: Don’t dumb it down: The effects of jargon in COVID-19 crisis communication
Source: PLoS One. 2020 Oct 7;15(10):e0239524. doi: 10.1371/journal.pone.0239524 (PMC7540871; doi:10.1371/journal.pone.0239524)
Supplement: S1 Appendix — An asterisk next to an item indicates the item was removed to improve reliability. (DOCX) [file pone.0239524.s003.docx]

**S1 Appendix. Items for All Scales Used in Analyses.**

*All response scales will be 7-point Likert scales.*

Processing Fluency Scale (Shulman & Sweitzer, 2018)

- The information presented felt new to me. (Reverse coded)
- Overall, I found reading this passage to be difficult. (Reverse coded)
- It was easy for me to understand the information presented.
- The passage felt easy to read.
- The information felt hard to read. (Reverse coded)
- The information felt like it took a long time to read. (Reverse coded)

Motivated Resistance to Persuasion (Nisbet et al., 2015)

- The message I saw was very objective. (Reverse coded)
- The message I saw tried to pressure me to think a certain way.
- The message I saw did not try to force its opinions on me. (Reverse coded)
- The message I saw was very believable. (Reverse coded)
- The message I saw was not very credible.
- The message I saw tried to manipulate me.
- Sometimes I wanted to "argue back" against message I saw.
- I found myself thinking of ways I disagreed with the message I saw.

Credibility (Appelman & Sundar, 2016)

- The message I saw seemed accurate.
- The message I saw seemed authentic.
- The message I saw seemed believable.
- The message I saw seemed trustworthy. (item added for this study)

Risk-Scale (Kahan et al., 2007)

- [TOPIC] pose a serious risk to human health.
- [TOPIC] pose a serious risk to human safety.
- [TOPIC] pose a serious risk to human prosperity.

Severity (de Zwart et al., 2009)

- How serious would it be for you if you got [TOPIC] in the next three months?
- How likely do you think it is that you will contract/experience [TOPIC] in the next three months?
- How likely do you think it is that you will contract/experience [TOPIC] in the next three months compared to other [women/men] of your age in the USA?
- [TOPIC] is something that will never happen to me.*
- I have a greater chance of [TOPIC] than most people
- My chances of contracting/experiencing [TOPIC] are very high.

* Item was removed to improve reliability

**References**

Appelman, A., & Sundar, S. (2016). Measuring message credibility: Construction and validation of an

exclusive scale. *Journalism & Mass Communication Quarterly, 93(1)*, 59-79.

de Zwart, O. et al. (2009). Perceived threat, risk perception, and efficacy beliefs related to SARS and

other (emerging) infectious diseases: Results of an international survey. *International Journal of*

*Behavioral Medicine, 16*, 30-40.

Kahan, D. M., Braman, D., Gastil, J., Slovic, P., & Mertz, C. K. (2007). Culture and identity‐protective

cognition: Explaining the white‐male effect in risk perception. *Journal of Empirical Legal Studies*, *4*(3),

465–505.

Nisbet, E.C., Cooper, K.E., Garrett, R.K., 2015. The partisan brain: How dissonant science messages lead conservatives and liberals to (dis)trust science. Ann. Am. Acad. Pol. Soc. Sci. 658, 36–66.

Shulman, H. C., & Sweitzer, M. D. (2018). Varying metacognition through public opinion questions:

How language can affect political engagement. *Journal of Language and Social Psychology*, *37*(2), 224

237.
