# Supplementary material for: Don’t dumb it down: The effects of jargon in COVID-19 crisis communication
Source: PLoS One. 2020 Oct 7;15(10):e0239524. doi: 10.1371/journal.pone.0239524 (PMC7540871; doi:10.1371/journal.pone.0239524)
Supplement: Attachment — Submitted filename: Response to Reviewers.docx [file pone.0239524.s004.docx]

Dear Dr. Holtgraves:

Thank you for your consideration of our paper (PONE-D-20-18585), entitled “Don’t dumb it down: The effects of jargon in COVID-19 crisis communication,” for publication in *PLOS ONE.* We are grateful for the opportunity to revise and resubmit the manuscript based on reviewer comments.

The suggestions conveyed by the reviewers and editor have prompted us to clarify the manuscript and more fully express our ideas and contributions. We believe these insights have considerably improved our paper.

In the following pages we point out the changes made, or responses to, specific reviewer comments by inserting them in red into the text of the previous correspondence. All changes to the original manuscript were inserted using track changes to make our updates more apparent. The line numbers and page numbers referenced in this response letter refer to the manuscript draft with track changes included.

Thank you again for your time and careful consideration.

PONE-D-20-18585
Don't dumb it down: The effects of jargon in COVID-19 crisis communication
PLOS ONE

Thank you for submitting your manuscript to PLOS ONE.  Although the research reported in this manuscript is important and interesting and has merit, we feel that it does not fully meet PLOS ONE’s publication criteria as it currently stands. Therefore, we invite you to submit a revised version of the manuscript that addresses the points raised during the review process. The primary concerns are summarized below; additional issues can be seen in the reviewers’ comments. A revision would need to address all of these (or provide a justification for not addressing).

We greatly appreciate the opportunity to revise our manuscript. We hope that the changes made sufficiently address all points.

Major issues:

First, as noted by Reviewers 1 and 3, there are confounds for your urgency variable in that the urgent message differs from the less urgent messages in multiple ways (familiarity, salience, personalization, etc.).  This is a serious limitation that should be noted, and your conclusions tempered accordingly.

We now include a thorough discussion of this concern in the newly added Limitations section of the paper (page 11, line 324). We also qualified our argumentation in the Discussion section when discussing our study conclusion and implications. We hope these qualifications address these concerns and help convey our findings more accurately.

Second, power is not mentioned until the Discussion when it’s noted that the study involved a smaller-than-intended sample size. The power and/or sample size considerations should be noted in the Method section and the criteria for describing the study as under-powered should be made explicit.

We should have been clearer in the previous version of the manuscript about the criteria we were using when making claims that our study was underpowered. We did not make those claims based on a formal a-priori or post-hoc power analysis. Instead, we made this inference based on the size of the estimates, the corresponding p-values, and the confidence interval locations in the flooding condition. A look at most of the confidence intervals surrounding the direct effects (Table 1), the indirect effects, and conditional estimates (Table 2) show confidence intervals that have one value at or around zero. Our thinking was that, with more power, some of the estimates that were very close to being below *p* < .05 (or barely encompassing zero in the confidence intervals), could be moved towards significance, or a non-zero estimate, with a slightly larger sample size and accordingly less error. These were the assumptions used to make the claim that we were underpowered, which, admittedly, is not the same criteria or rigor as a formal power analysis.

In this revision, we have removed our discussion of power, given that we did not run a formal a-priori analysis. Instead, we used our discussion of study limitations to focus on the concerns raised in this review, which seemed more relevant to accurately interpreting study conclusions. That said, if the editor would like us to conduct post-hoc power analyses and report these in our paper, we are happy to do so.

Third, the sample consists of MTurk workers and so there should be some mention of the limitations (e.g., non-naivety and trustworthiness) of participants from this platform.

Done. The drawbacks of MTurk workers have now been acknowledged in the limitation section. (page 12, line 355). Furthermore, given known data integrity issues related to MTurk, we now extend our discussion in the Method section to clarify our selection criteria. Specifically, we made sure that all participants could pass a CAPTCHA, pass an attention check, respond to an open-ended question, and had a 95% completion rate on over 500 HITS (page 5, line 176).

Fourth, the analyses are presented in a clear and straightforward manner.  However, readers would likely appreciate more descriptive data (means and standard deviations) regarding your critical variables (e.g., fluency as a function of jargon and urgency; but see also Reviewer 1’s request for means and standard deviations for your outcome variables); if not presented in the text they could be included in your supplementary files.

Good suggestion. Conditional means and standard deviations for all study variables are now included in a supplementary file labeled S3.

Please include the following items when submitting your revised manuscript:

- An unmarked version of your revised paper without tracked changes. You should upload this as a separate file labeled 'Manuscript'.

Kind regards,

Thomas Holtgraves, Ph.D.
Academic Editor
PLOS ONE

------------------------

Journal Requirements:

When submitting your revision, we need you to address these additional requirements.

1. Please ensure that your manuscript meets PLOS ONE's style requirements, including those for file naming. The PLOS ONE style templates can be found at

[https://journals.plos.org/plosone/s/file?id=wjVg/PLOSOne_formatting_sample_main_body.pdf](https://urldefense.com/v3/__https:/journals.plos.org/plosone/s/file?id=wjVg*PLOSOne_formatting_sample_main_body.pdf__;Lw!!KGKeukY!ltoOUCnceVfTdqZVFiuGyCxx1ja1Bt_Ow_XOq_XkxLmj2jrQ_wkYuBd0o74B6WVytQ$) and

[https://journals.plos.org/plosone/s/file?id=ba62/PLOSOne_formatting_sample_title_authors_affiliations.pdf](https://urldefense.com/v3/__https:/journals.plos.org/plosone/s/file?id=ba62*PLOSOne_formatting_sample_title_authors_affiliations.pdf__;Lw!!KGKeukY!ltoOUCnceVfTdqZVFiuGyCxx1ja1Bt_Ow_XOq_XkxLmj2jrQ_wkYuBd0o75hBZWssw$)

We believe that all of our files meet these requirements.

2. Please provide additional details regarding participant consent. In the Methods section, please ensure that you have specified (1) whether consent was informed and (2) what type you obtained (for instance, written or verbal). If your study included minors, state whether you obtained consent from parents or guardians. If the need for consent was waived by the ethics committee, please include this information.

Done. The consent process has now been explained on page 6, line 207. We describe that the first page of the study included an IRB approved consent form that stated by clicking ‘proceed’, participants were providing their consent to participate in the study.

3. Thank you for stating the following financial disclosure:

"No. The funders had no role in study design, data collection and analysis, decision to publish, or preparation of the manuscript."

At this time, please address the following queries:

a. Please clarify the sources of funding (financial or material support) for your study. List the grants or organizations that supported your study, including funding received from your institution.

Done. A statement indicating that this work was funded through our institution has now been added to the cover letter.

b. State what role the funders took in the study. If the funders had no role in your study, please state: “The funders had no role in study design, data collection and analysis, decision to publish, or preparation of the manuscript.”

The language suggested (“The funders had no role in study design, data collection and analysis, decision to publish, or preparation of the manuscript.”) has now been included in the cover letter.

c. If any authors received a salary from any of your funders, please state which authors and which funders.

Not Applicable.

d. If you did not receive any funding for this study, please state: “The authors received no specific funding for this work.”

Not Applicable.

Please include your amended statements within your cover letter; we will change the online submission form on your behalf.

Done. Statements related to points A and B have been added to the cover letter.

4. We note that you have stated that you will provide repository information for your data at acceptance. Should your manuscript be accepted for publication, we will hold it until you provide the relevant accession numbers or DOIs necessary to access your data. If you wish to make changes to your Data Availability statement, please describe these changes in your cover letter and we will update your Data Availability statement to reflect the information you provide.

No changes need to be made to our previous statement. Our data is already available on the OSF link provided in the manuscript.

[Note: HTML markup is below. Please do not edit.]

Reviewers' comments:

Reviewer's Responses to Questions

**Comments to the Author**

1. Is the manuscript technically sound, and do the data support the conclusions?

The manuscript must describe a technically sound piece of scientific research with data that supports the conclusions. Experiments must have been conducted rigorously, with appropriate controls, replication, and sample sizes. The conclusions must be drawn appropriately based on the data presented.

Reviewer #1: Yes

Reviewer #2: Partly

Reviewer #3: Partly

2. Has the statistical analysis been performed appropriately and rigorously?

Reviewer #1: Yes

Reviewer #2: Yes

Reviewer #3: Yes

3. Have the authors made all data underlying the findings in their manuscript fully available?

The [PLOS Data policy](https://urldefense.com/v3/__http:/www.plosone.org/static/policies.action*sharing__;Iw!!KGKeukY!ltoOUCnceVfTdqZVFiuGyCxx1ja1Bt_Ow_XOq_XkxLmj2jrQ_wkYuBd0o74j6uLwQA$) requires authors to make all data underlying the findings described in their manuscript fully available without restriction, with rare exception (please refer to the Data Availability Statement in the manuscript PDF file). The data should be provided as part of the manuscript or its supporting information, or deposited to a public repository. For example, in addition to summary statistics, the data points behind means, medians and variance measures should be available. If there are restrictions on publicly sharing data—e.g. participant privacy or use of data from a third party—those must be specified.

Reviewer #1: Yes

Reviewer #2: No

Reviewer #3: Yes

4. Is the manuscript presented in an intelligible fashion and written in standard English?

PLOS ONE does not copyedit accepted manuscripts, so the language in submitted articles must be clear, correct, and unambiguous. Any typographical or grammatical errors should be corrected at revision, so please note any specific errors here.

Reviewer #1: Yes

Reviewer #2: Yes

Reviewer #3: Yes

Reviewer #1: Review of PONE-D-20-18585: Don't dumb it down: The effects of jargon in COVID-19 crisis communication.

The manuscript presents a study that examined the effect of jargon on resistance, reactance, credibility, and perceived risk across three different topics associated with differing levels of urgency. Whereas jargon use affected processing of information associated with low urgency, jargon use has no discernible influence for high-urgency information (compared to a high urgency control information).

Overall, the paper is well written and organized. The authors address an interesting and timely topic.

Thank you.

The prediction that increased processing from increased motivation or ability has been borne out in many studies. In most cases, these predictions focus on differences in an outcome (e.g., resistance, attitude change, etc.). With jargon having no difference in resistance and credibility in the high urgency message, it implies that, from a practical standpoint, jargon is not needed for a high urgency message to be effective. Is this the case?

Yes. Put a different way, our findings suggest that the presence or absence of jargon does not affect how people evaluate high urgency messages. This implies that when people are motivated to process information (even when that information is complex), then features of the message style (such as jargon) exert a lesser influence than when people are not motivated to process information. We agree that links between processing and motivation and/or ability have been borne out in many studies (and now say so on page 12, line 376), but we hope that the ecological link made here, which uses crisis communication during an ongoing, and novel, public health crisis, provides another practical demonstration of this psychological process.

Another question the authors may want to address relates to the urgency and jargon manipulations. Specifically, the issue is different across levels of the manipulation. This is reasonable, but one can imagine that the difference in topic also affects other variables (e.g., experience with topic, salience via news exposure, etc.). Importantly, the jargon would also differ across the topics. Perhaps the authors could speak to that.

These are all good points. Given that the substantive information necessarily differed by topic condition, we agree that the messages probably varied in ways beyond urgency and jargon. We now acknowledge this limitation in the paper (page 11, line 327) and suggest a message sampling approach for future investigations into this topic. That said, in our effort to make the messaging between topics as consistent as (reasonably) possible, the second half of our stimulus message (conveying guidelines for dealing with emergencies/risk) was the same across all topic conditions. This way, at least the last experimental message that participants were exposed to was the same. We hope that this decision reduced some of the possible unintended message variance between topics. We now highlight this message equivalency consideration when we discuss our stimuli in the paper (page 7, line 224).

Another question I have relates to the conceptualization of counterarguing. The term (which refers to actively refuting claims made in the message) seems to be used a catch-all phrase for thought-based resistance. But, in reality, there are different forms of resistance beyond counterarguing (e.g., thought bolstering, source derogation, etc.). That said, when describing the research on Page 4, line 70 (references 4 & 11), is it the case that participants counterargued. In other words, did they really counterargue under low processing? From reading the description of the materials on page, the general term of resist would be more consistent with what is being measured and what participants did.

Agreed. The mention of counterarguing referenced here has now been changed to *resist* for accuracy and consistency as this reviewer points out (page 3, line 78). Thank you for this suggestion.

The authors are predicting interactions on the relevant outcome variables. If space allows, it would be nice to see those results and subsequent means and standard deviations.

Good idea. We now include a table in the supplementary materials (S3) that includes all of this information.

My apologies, but it is unclear how the analyses in the mediational model were conducted with three levels of the topic/urgency variable. Can the authors clarify how this is handled?

We used COVID-19 as the referent category for these tests, and then tested the differences between the policy issue and flood issue against this category for the impact of topic (and jargon) on processing fluency (Table 1). More specifically, the macro PROCESS (Hayes, 2013) includes an option for categorial variables in the model. Just like in a multiple regression analysis, the remaining two topics were compared against the referent category (in our case, COVID-19). A non-zero difference indicates that, when controlling for other factors, there is a significant difference between these conditions. The statistical model for this analysis is depicted in Figure 1 and each path maps onto the estimates provided in Table 1. Additionally, an interaction effect between topic conditions and jargon was included, and the conditional effects were presented that indicate moderation between topic and jargon conditions consistent with our predictions (jargon condition exerts a significant non-zero influence on processing fluency [the model mediator] in the flood and policy topic condition, but exerts a non-significant influence on processing fluency in the COVID condition. All of these estimates are provided in Table 1.

Reviewer #2: 1. Is this manuscript technically sound, and do the data support the conclusions?
Yes, to both except: Provide one sentence on how random assignment was accomplished

A sentence that states random assignment was accomplished using the randomizer function in Qualtrics has now been included on page 6, line 202.

and what was the N per cell.

The *N’s* per cell varied from 62-67 people per condition. The exact numbers are now included in the newly added Table S3, which reports descriptive statistics for all conditions and outcome variables.

Also, it is not clear why jargon should influence processing fluency (lines 119, 120). It would be useful to elaborate on this. An individual may work harder in trying to understand an important message but why would this affect fluency?

We are happy to elaborate on this idea as it is one of the more important points of our manuscript. Specifically, as the reviewer brings up here, the presence or absence of jargon may affect how “hard” a person has to work when trying to understand a message. This idea of something requiring effort to understand is the feeling conceptualized by processing fluency. Processing fluency refers to the amount of work people *feel* like they are putting in during message processing. And, as we argue, these feelings of effort affect how people evaluate the issue (beyond simply the content of a message) at hand. In an effort to make this link clearer, we have actually included this reviewer’s language in our paper (page 3, line 88), to underscore the point that processing fluency reflects how hard someone *feels they have to work* to understand a message. We think this language is an effective way to think about this concept, and thank the reviewer for sharing it with us.

Also, a 2 X 3 table showing fluency scores would help in the results.

Done. A table that includes descriptive statistics across all measures, by condition, is now included in the supplementary materials as S3.

2. Has the statistical analysis been performed appropriately and rigorously?
The results came out nicely. However, for the DV of fluency would it be helpful to determine if the regression coefficients for policy topic (B = -.74) versus the flood topic (B = -.47) were different? If so, it would tie in nicely with the Figure 1 result.

Depending on the model, sometimes these issues significantly differed from one another, but often they did not. To make this assessment, rather than run significance tests across all possible comparisons, we offer 95% confidence intervals for all estimates. When the intervals overlap, this could be interpreted as a “non-significant” differences between estimates.

That said, by presenting confidence intervals instead of only significance tests, we give the reader more information. For example, confidence intervals provide interested readers with more nuanced interpretation regarding the size of these estimates, the degree to which each estimate overlaps with others, and the relative strength of each estimate. As shown with both the direct (Table 1) and indirect (Table 2) effects of topic on processing fluency, there is a “significant” difference between the COVID condition and the Policy condition more often than not. The flooding condition always sits in the middle of both estimates and usually overlaps with both. However, we do not think this overlap is problematic because, when looking at the confidence intervals across all topic conditions, the trend is always the same. This is why we opted to present Figure 2 to help illustrate this pattern of findings. If one were to interpret these findings categorically (significant/not) some of the nuance would be missed with regard to the pattern of findings overall. Findings are always very weak (and near zero) in the case of the COVID condition, slightly stronger for flooding, and strongest for the policy condition. In order to best illustrate these findings, we present confidence intervals and Figure 2. We hope this presentation of results now makes more sense and provides the reader with more information, not less.

3. Have the authors made all data underlying the finding in the manuscript fully available?
I could not find any data. I found the 6 different scripts used in the experiment. I also found the five ratings scale employed.

We have since re-uploaded this dataset, including all of the newly requested covariate measures, to the [open science framework](https://osf.io/gb5nc/?view_only=4252a3c254524b6c9521cecbbd9e4591). We have also since double-checked to make sure the dataset is clearly labeled under the *Materials* tab. Hopefully, this data is now easier to find.

4. Is the MS presented in an intelligible fashion and written in standard English?
OK but tighten up the sentences; read MS out loud.

Grammatical changes have been made throughout in an attempt to reduce wordiness where possible.

Reviewer #3: The question posed is interesting, the theory and literature considered as background was appropriate, and the article is written in a crisp, clear style. Non-specialists could read it and appreciate the main claims, but would find it more difficult to follow the analyses.

Thank you for your comments. Though mediational analyses are not always intuitive, we have made changes to improve the interpretability of our findings, including adding conditional descriptive statistics coupled with the trends displayed in Figure 2.

My biggest concern is the conclusion. It seems too strong and should be modified.

This is a fair concern and consistent with what others have noted. To this end, we have since qualified some of our original statements. We also extended and emphasized study limitations by creating a new section that acknowledges the concerns raised here. We hope these edits help convey our findings more accurately.

The independent variable of interest is "urgency" and whether this affects the usual finding that jargon reduces the effectiveness of public policy recommendations. The problem with any singular sampling of topics differing in urgency, especially given that one of those topics was chosen because of its time sensitivity, is that it is always the case that the communications will differ in ways other than the variable of interest (i.e., urgency). COVID is primarily an individual, personal threat, while floods are more regional, less personal, and more of a threat to property. The policy (no urgency) communication is more abstract, as well as less urgent. More people know something about disease dynamics than about hydraulics and geology of flooding. The punchline is that a conclusion claiming that concern about jargon may be less pressing for urgent communications, and might even increase the persuasiveness of the communication, seems too strong a claim to draw from a single set of communications that vary in a number of ways besides urgency.

Agreed. These are all strong points. We hope that our extended discussion of this limitation, including some of the ideas brought up here, offers a more tempered portrayal of our findings. Additionally, we removed the claim speculating about the positive effects of jargon guided by this advice.

Another possible threat to internal validity is that respondents had been exposed to a considerable number of expert descriptions of the COVID threat, so that they had some familiarity with the COVID style jargon, in a way that was not true of flooding and disaster policy concerns. None of the concerns I am raising are disqualifying in my opinion, but the author should be more muted in characterizing the conclusions, or indicating limitations of the study.

We now mention the possibility of differential familiarity with the jargon terms (for COVID-19 in particular) in the limitations section (page 11, line 332). That said, although we acknowledge that the jargon terms might have been more familiar in the COVID condition relative to the other conditions, interestingly, we do not think this possibility undermines our primary argument. More specifically, we argue that when people are motivated to process information, they will be more focused on message substance than style. If people ended up learning these complicated terms due to the threat/urgency of COVID, then our primary conceit – that experts do not need to simplify language in a crisis – remains supported. Although these ideas are possible, they are currently just speculation on our part, and we hope to address these gaps in future work. In the meantime, we did not include this rationale in the paper but bring it up in our response to this reviewer because we thought it was an interesting idea to consider.

Minor: (1) Why was the "never happens to me" question removed? How did it affect reliability?

We used Cronbach’s alpha to determine reliability for all our scales. For this scale in particular, the alpha value with all measures was .789. When this item was removed, the alpha jumped up to .87. We thought that a difference of .08 seemed to justify the removal of this item. That said, we made responses to all items across all scales available in the dataset posted on OSF.

(2) Why was there no measure of urgency, or were the Risk questions just that? If so, the differences in urgency are not as strong as would be desirable?

We regret that we did not have a more precise manipulation check measure in our design, especially after the thoughtful comments raised here regarding possible differences between messages across topic condition. Also, we agree, that the differences in urgency (based on the Risk questions at least) were not as strong as we’d hoped. That said, the fact that we obtained significant effects across multiple measures, despite a relatively weak manipulation, offers encouraging support for the existence of the relationships we observed. We would hope that a stronger, and cleaner, manipulation should lead to even stronger effects. This is something we are working towards in future work, alongside the creation of a better manipulation check measure.

(3) It's too bad that the regions of the country where the respondents lived were not known? The COVID threat at the time was centered in the Northeast. Similarly, flood risk varies enormously by where one lives. Questions on prior exposure to flu, floods, other disasters, would have been interesting to include.

We actually have this information and now include these variables on OSF. Specifically, we asked a) state of residence, b) whether the participant had ever experienced a flood [yes/no], and c) whether they had ever tested positive for COVID [yes/no]. We did not ultimately use this data because, given the experimental design, we did not really have enough people from one state (or even region), or who responded affirmatively to these questions, to make meaningful comparisons across conditions.

For example, in the case of State, the state with the highest frequency of participants was California (N = 42), but, even in this case, this would only amount to 7 people per condition. Although we could decide to aggregate people by region, making the decision for regional groupings on top of risk associated with these regions, would be difficult to do with a high level of objectivity. Additionally, although regional splits may provide more data per cell, at the time of this data collection, COVID cases were isolated to specific areas (for example, New York City more so than New York state) or specific suburbs (outside Seattle), so this adds some noise as well. And, on top of this, states were dealing with COVID in dramatically different ways that should affect urgency perceptions, even though case numbers were low. For example, at the time of this survey, California was effectively shut down before a high number of cases occurred. This made it difficult for us to figure out ahead of time whether CA perceptions would be more similar to NY, due to similarly bold state government responses, or whether they would differ due to very different caseloads. In sum, given all these issues and complexities, we couldn’t figure out how to use this variable effectively even though contextual urgency should certainly play a role in the relationships under investigation.

The same was true with regard to those who had experienced a flood (n = 76) and tested positive for COVID (n = 40). When looking at the number of affirmative cases across conditions, we did not see how we could draw any meaningful conclusions with the limited data. We do agree, however, that these covariates would likely affect our outcomes in interesting ways and wish we had more data to find out how.

(4) the different priced samples (80 vs. 200) could be viewed as reflecting a difference in motivation with the 80 cent group the more motivated to attend to communications.

This is possible. Differences in motivation could certainly be associated with the size of the incentive in either direction one could imagine. We now state this possibility in the paper on page 12, line 367.
