# Supplementary material for: Don’t dumb it down: The effects of jargon in COVID-19 crisis communication
Source: PLoS One. 2020 Oct 7;15(10):e0239524. doi: 10.1371/journal.pone.0239524 (PMC7540871; doi:10.1371/journal.pone.0239524)
Supplement: Attachment — Submitted filename: Response to Reviewers.docx [file pone.0239524.s005.docx]

Dear Dr. Holtgraves:

Thank you for the conditional acceptance of our paper (PONE-D-20-18585R1), entitled “Don’t dumb it down: The effects of jargon in COVID-19 crisis communication,” for publication in *PLOS ONE.* We are grateful for the opportunity to publish in this outlet, pending of course your approval of the minor revisions listed below.

Thank you again for your time and careful consideration throughout this process, especially during this chaotic time. This was a great experience.

Respectfully,

Hillary Shulman and Olivia Bullock

PONE-D-20-18585R1
Don't dumb it down: The effects of jargon in COVID-19 crisis communication
PLOS ONE

Dear Dr. Shulman,

I am writing with regard to your manuscript, "Don't dumb it down: The effects of jargon in COVID-19 crisis communication" (PONE-D-20-18585R1). I have read your revision, and find that you have done a nice job addressing the concerns raised in the first round of reviews. As such, I am taking the action of accepting your manuscript pending some very minor revisions.

There are three small issues that I would like you to address in your revision:

1 -- I think it would be helpful if you could make your data available as excel spreadsheets in addition to the SPSS files. The excel sheets will probably be more accessible to more users than the SPSS files. In addition, please upload your output file as a PDF so that those without SPSS will have access.

Good suggestions. An excel file that includes all pertinent data has now been posted on OSF, as well as an up to date version of the results output (see below) and a pdf version of this output.

2 -- Please proof carefully the data presented in the manuscript. Just spot checking I found one error (the MRTP constant is 5.61 in the output but reported as 5.86 in Table 2).

Thank you for double-checking these numbers. Just to be on the safe side, we re-ran all of our analyses, updated all Tables, and posted these up to date results on OSF. We also double-checked to ensure that the information from the OSF output perfectly matches the information in the manuscript and now believe that all inconsistencies have been corrected.

3 -- In studies like this I believe it is useful to provide readers with the experimental cover story (i.e., instructions for the participants). These could be provided in your supplementary materials.

No problem. We added this information to the supplementary file (S1_File) that contains the experimental messages used. These instructions read, “On the next three pages, you will see information about national emergencies. Please read the information carefully. You will not be permitted to advance past each page until you have read the information.”

Once you've addressed these final points, I will take a quick look and send you the official acceptance letter.

Thank you again for all your help and guidance on this manuscript!

If you would like to make changes to your financial disclosure, please include your updated statement in your cover letter. Guidelines for resubmitting your figure files are available below the reviewer comments at the end of this letter.

No changes are needed

If applicable, we recommend that you deposit your laboratory protocols in [protocols.io](http://protocols.io/) to enhance the reproducibility of your results. Protocols.io assigns your protocol its own identifier (DOI) so that it can be cited independently in the future. For instructions see: [http://journals.plos.org/plosone/s/submission-guidelines#loc-laboratory-protocols](https://urldefense.com/v3/__http:/journals.plos.org/plosone/s/submission-guidelines*loc-laboratory-protocols__;Iw!!KGKeukY!g0tGkG_7yYYrY0M4xsVnCQBSL-EmMMcF9VPBEBDzWpJyXYfflCCHcvSg_xSKIjRvmg$)

N/A

We look forward to receiving your revised manuscript.

Kind regards,

Thomas Holtgraves, Ph.D.
Academic Editor
PLOS ONE
